# Training in Clinical Research in India: Potential and Challenges

**DOI:** 10.4103/0970-0218.55267

**Published:** 2009-07

**Authors:** Sanjay P Zodpey, Himanshu N Negandhi

**Affiliations:** Public Health Foundation of India, New Delhi, India

Clinical Research is a branch of medical science that determines the safety and effectiveness of medications, devices, diagnostic products, and treatment regimens intended for human use. These may be used for prevention, treatment, diagnosis, or for relief of symptoms in a disease.(1) The rapid momentum in the growth of clinical research over the last decade is expected to continue in the near future. This rapid growth is also an attractive business opportunity, with an estimated business worth over US $ 1.5 – 2 billion,(2) with the country receiving increasing attention for the conduction of clinical research.(3) Clinical research is a vital and essential piece in this market jigsaw before the introduction of a new drug or vaccine.

Clinical research is not a modern occurrence with the earliest evidences of clinical trials in the writings of Avicenna.(4) He elucidated the rules and principles for testing the effectiveness of new drugs and medications, which forms the basis of clinical pharmacology and clinical trials. One of the well known clinical trials recorded in history was conducted by James Lind aboard the HMS Salisbury.(5) Lind attempted to scientifically evaluate various treatment regimes for the control of scurvy, a major problem affecting sailors during that period. Several other comparative studies were done in the eighteenth and nineteenth centuries. It was in 1926 that Fischer introduced the concept of randomization in agricultural studies.(6) The first human clinical study with randomization was done by Amberson in 1931.(7) He carefully matched 24 patients of pulmonary TB with comparable groups of 12 each with the flip of a coin. This was the first example of the use of randomization for assignment of study participants into groups. The MRC trial for streptomycin was the first trial to use random numbers for allocation to study and control groups.(8) With the emergence of clinical trials as the preferred method for the evaluation of clinical interventions in the last 30 years, the techniques of implementation and analysis have shown rapid progress. The progress has been aided by technological advances in computers and information technology. The reporting of trials has also gained prominence with increasing attention toward this aspect of work. The macro environment for the conduction of clinical research in India is now receiving due recognition, with the publication of our own good clinical practice (GCP) guidelines,(9) and the establishment of a clinical trials registry.(10) However, the facilitation of clinical research also needs the presence of highly trained manpower. Although to date, little constructive debate has been focused on the scenario of clinical research training needs.

Training institutes in clinical research have mushroomed throughout the country to reap the benefits of urgent manpower requirements of the industry. The rapid growth of the clinical research field has further encouraged this growth in the past three years. The emergence of highly task-specific responsibilities like Pharmacovigilance has also increased the complexity of training institutes. Clinical research is a wide field; a basic dichotomy is evident between jobs related to scientific conduction and reporting of trial results on the one hand; and the operational issues related to the trial conduction on the other hand. Both sections are mutually exclusive during recruitment, but need staff that is highly complementary to each other's role in the team. This dichotomy in the roles played after recruitment, mandates differing skill-sets for the professionals in clinical research. The basic skill-sets expected by the industry could be acquired as a part of the academic background, prior experience with the industry, or specific training/education in clinical research. Current recruitment is highly influenced by prior work-experience with the clinical research industry. Almost all the courses that are offered cater to the provision of foot-soldiers to the clinical research industry. The operations skills that are imparted to the course participants are the bare minimum, to gain an entry into the clinical research profession. Further career advancement is then through routine promotional avenues within the system and are not linked to the training course.

There are no government run institutes offering training in clinical research in India. In the absence of a formal university structure, UGC/AICTE recognition, or recognition under any state act, the courses that are offered do not have a formal Indian University accreditation. The eligibility criteria for these courses are consequently wide, with post-doctoral students and paramedical graduates being enrolled for the same class. Weekend programs offering a quick entry into the field are in vogue. Such programs offer little hands-on experience to the potential entrants into clinical research. Fancy names promising a fruitful job and high salaries abound the newspapers.

The current training scenario poses the question of the *quantity* of trained product as well as the hidden need for improving the *quality* of training. Training activities need to be formally instituted for Institutional Ethics Committee members, investigators, and officials currently engaged in clinical research. At the same time, there is an urgent need for capacity building for new trainees as well. The availability of qualified professionals who can skillfully plan, execute, monitor, and fully assess the dimensions of their work is an urgent necessity. The current output from the training institutes is unable to provide personnel for responsibilities across the hierarchy in clinical trial conduction. The science of clinical trial is a wide and highly specialized field. Research strength in clinical research is not demonstrated just by the revenue flow or the number of research projects undertaken in a given year. The innovative and scientific spirit culminating in the conduction of genuine Indian-designed research would be an accurate measure of the research strength. Concerted efforts are needed to address this vexing issue.

Promotion of clinical research warrants a wider consensus among the diverse stakeholders. Protection of patient interest and ethical issues are paramount and genuine concerns. Institute Ethics Committee (IEC) strengthening efforts and compulsory trial registration could be undertaken in the immediate future. The standard number of hours of teaching for granting a diploma/degree needs to be formalized as a part of the essentials in the clinical research curriculum. A structured curriculum that is responsive to modern day issues would find great support. Research activities with wider Indian public health significance could be prioritized and fast-tracked. This could include exploring the role of traditional Indian medicines for health benefits. The formulation of accreditation criteria for clinical research trainings could be contemplated as a medium-term strategy. The future Indian foray into clinical research will be highly dependent on the Indian ability to design indigenous research protocols. A time-bound activity to address the generation of this specialist manpower could be contemplated at various public health fora. Inter-institutional partnerships for research conduction and research funding could offer quick but temporary solutions.

The current bright outlook of the clinical research market is not a research utopia. Significant time and attention will be needed to address the concerns of the Indian scientific community and independent researchers. If unanswered, these could be potential deterrents in the rapid growth of clinical research in India.
